# Supplementary material for: Permanent Makeup Removal Severe Complication—Case Report and Proposal of the Protocol for Its Management
Source: J Clin Med. 2024 Sep 22;13(18):5613. doi: 10.3390/jcm13185613 (PMC11432740; doi:10.3390/jcm13185613)
Supplement: Supplementary file 1 [file jcm-13-05613-s001.zip › jcm-3142699-supplementary.pdf]

Safety Data Sheet

in accordance with EC Directive 1907/2006/EG , Artikel 31

----

1. Identification of the substance / preparation and company

Long-Time-Liner Conture Make up GmbH

Tal 14 - 18

80331 München

Emergency Information:

Dr. Sherif Aly: **0049 89 2420 9019**

Dr.Köhler Consulting: **0049 6161 8067 115**

Product Name: **-Bye-Bye Pigment**
Created: Juni 2019

Only for Germany:
Poison Information Centre- Mainz: 06131-19240 Göttingen: 0551-19240 Erfurt: 0631-730730

----

2. Hazards_Identification

Aqueous, ethanolic and alkalic solution for a chemical extraction of colour pigments.

Ethanol 96% CAS-Nr.: 64-17-5 GHS 02;GHS 07 ; SW: Danger
H 225; H 319, P 210; P 233

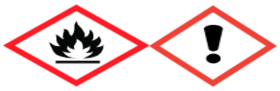

Zink Oxide CAS-Nr.:1314-13-2 GHS 09; SW: Warning, H 410; P 273

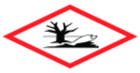

Calcium Hydroxide 71% CAS: 1305-62-0 GHS 05, GHS 07; SW: Danger
H 290, H 301, H 317, P 260; P 280

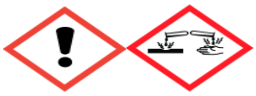

Sodium Hydroxide CAS: 1310-73-2 GHS 05; SW: Danger; H 290, H 314

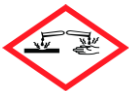

P 303 + P 361 + P 353; P 305+ P 351+ P 338;

3.Commposition/information on ingredients

High flammable Liquid; May cause great eye irritation. May cause skin irritation; Keep away from children.

Safety Data Sheet
-Bye-Bye Pigment

4. First Aid Measures:
Never give fluids or induce vomiting if injured person is unconscious or having convulsions.

After skin contact:
Wash off in the shower or under running water.

After eye contact:
Immediately rinse thoroughly under running water.

After swallowing:
Consult an physician. He will decide on need and manner of gastric emptying.

5. Fire Fighting Measures
Suitable extinguishing media:
Water fog or fine spray. Carbon dioxide, chemical foam. Water jet.

For safety reasons unsuitable extinguishing agents:
No

Hazardous Combustion Products:
Sulfur dioxide, nitrous gases,
Carbon monoxide during incomplete combustion

Special protective equipment for fire-fighters:
Approved positive pressure self-contained breathing apparatus
Wear suitable respiratory equipment and carry fire fighting clothing.

Special hazards arising from the substance or preparation itself:
Not known.

6. Accidental Release Measures
Methods for cleaning up / taking up
Collect the product with shovels or suckers from the ground. Dispose of according to applicable rules and regulations.

Soak up with absorbent paper.
Product can chemically dissolve some surfaces on the floor.

7. Handling and Storage
Handling
Product can build up on the floor slippery surfaces. Protect the product from open fire.
Storage
Recommended storage temperature: 5-20 degree Celsius. Protect product from strong sunlight
Protect from frost.

8. Exposure controls and personal protection

Exposure Limits
None established

Components with workplace to be monitored:
No

Technical measures
Good general ventilation should be sufficient

Personal Protective Equipment
Protective gloves, non-slip shoes

Respiratory protection
Respiratory protection is not required

Skin Protection
Protective gloves and clean body-covering clothing

Eye / Face Protection
Eye Protection is not required but its better to wear safety glasses

9. Physical and chemical properties

Appearance: aquous and ethanolic solvent
Color: white suspension
Odour: not perfumed (comparison samples retained)

Boiling point: about 86 degrees Celsius
Freezing point: ca. -20 degrees Celsius
Solubility in water: very good

Vapour pressure: not applicable
Relative vapor density: not applicable
Specific Gravity: about 1,035 g / cc
pH: ca. 9,0 – 11,0
LogP (octanol / water): not applicable

Flash point: ca. 40°C
Ignition temperature: > 80 degrees Celsius
Lower explosion limit: not applicable
Upper explosion limit: not applicable

10. Stability and reactivity

Chemical Stability
Stable under normal handling and storage conditions, see Section 7,Handling and storage

---

Materials to avoid
Oxidant

11. Toxicological information

Swallow
Oral toxicity is not known.

Skin contact
Usually moderate skin irritant. Due to the physical properties of a skin absorption can not be excluded.

Eye contact
In general, irritating to the eyes. In case of contact with eyes, rinse immediately with a lot of water.

Inhale
Harmful effects not anticipated by inhalation.

Other Information
Based on the review of existing data are appreciable adverse effects
Not expected to be repeated exposure and conditions of use.
Pay close attention to the instructions for use.

12. Ecological Informations

Reduction
Biodegradability is in accordance with the legal provisions for detergents.
It goes from the product of no water hazard if it is sufficiently diluted.

Aquatic toxicity
The impact on aquatic systems has not been tested.

13 Disposal considerations

Dispose in accordance with all waste management related laws and regulations.
Since 01.01.1999 to the EC Directive 91/689 EEC.
For the correct coding and designation of expenses incurred him waste is the disposer responsible.

14. Transport Information

Classified as a dangerous good in the meaning of transport regulations in the EU and outside the EU.
UN-Nr.: 1824 Sodium Hydroxide Solution; Hazard Klass: 8; Pachaging Group : II
Since the packaging is smaller than 125 ml, the regulatios for small packaging apply.

15. Regulatory

EU Classification and Labelling

N Dangerous for the enviroment
H 225 Highly flammable liquid and vapor
H 290 May be korrosive to metals
H 301 Toxic if swallowed.
H 302 Harmful if swallowed.
H 304 May be if swallowed and enters airways fatal
H 312 Harmful in contact with skin
H 314 Causes severe skin burns and eye damage.
H 315 Causes skin irritation
H 317 May cause an allergic skin reaction
H 318 Causes serious eye damage
H 319 Causes serious eye irritation
H 335 May cause respiratory irritation
H 336 May cause drowsiness or dizziness
H 373 May cause damage to organs through prolonged or repeated exposure
H 400 Very toxic to aquatic life
H 410 Very toxic to aquatic life with long lasting effects.
H 411 Toxic to aquatic life with long lasting effect
P 210 Keep away from heat / sparks / open flames / hot surfaces. Do not smoke!
P 211 Not against an open flame or other ignition source
P 260 Do not brethe dust/fume, gas, mist, vapour spray
P 273 Avoid release to the environment.
P 280 Wear protective gloves/protective clothing/eye protection/face protection.
P 303 IF ON SKIN (or hair):
P 305 If in eyes:
P 314 Get medical advice/attention if you feel unwell
P 338 Remove contact lenses if present and easy to do. Continue rinsing.
P 351 Rinse cautiously with water for several minutes
P 353 Rinse skin with water [or shower].
P 361 Take off immediately all contaminated clothing.

GHS 05 Danger or Warning -Corrosive cat.1
GHS 06 Danger -Toxic ca. 1-3
GHS 07 Warning -Toxic cat.4, Irritant cat. 2 or 3, Lower systemic health hazards
GHS 08 Danger or Warning -Systemic health hazards
GHS 09 Warning (for cat.1) Enviroment

National regulations-Germany
-Wassergefährdungsklasse:
WGK 1 -VwVwS;

16. Other information

No further data

The information is based on current knowledge. They are our products
in terms of safety requirements describe and thus have not the meaning,
guarantee certain properties.
Existing laws and legislation are observed by the recipient of our products
to note responsibility.
